# Extending eFall risk prediction to working-age adults within mental health and learning disability services: a clinical validation study

**DOI:** 10.1038/s41598-026-51298-0

**Published:** 2026-08-10

**Authors:** Tianhua Chen, Luise V. Marino, Kate Best, Sean Bhatnagar-Knox, Victoria Humble, Michael Garnham, Karen Greenbank, Samuel Relton, Stephen Lim, Andrew Clegg

**Affiliations:** 1 School of Computing and Engineering, University of Huddersfield, Huddersfield, UK; 2 South West Yorkshire Partnership Teaching NHS Foundation Trust, Wakefield, UK; 3 School of Human and Health Sciences, University of Huddersfield, Huddersfield, UK; 4 Faculty of Medicine, University of Southampton, Southampton, UK; 5 Faculty of Medicine, University of Leeds, Leeds, UK; 6 NIHR Applied Research Collaboration Wessex, University Hospital Southampton NHS Foundation Trust, Southampton, UK

**Keywords:** Fall risk prediction, External validation, Electronic health records, Model recalibration, Mental health, Learning disability, Working-age adults, Logistic regression, Diseases, Health care, Medical research, Psychology, Psychology, Risk factors

## Abstract

Most fall risk tools are developed for older adults; evidence in working-age populations receiving mental health services is limited. To externally validate the existing *eFalls* prediction model in working-age adults (18–65 years) within a UK integrated teaching National Health Service (NHS) foundation trust and assess the impact of simple recalibration. We conducted a retrospective validation using routinely collected electronic health records from a large integrated teaching NHS foundation trust in the north of England. The published *eFalls* coefficients were applied to derive 12-month fall/fracture risk. Performance was evaluated using discrimination (C-statistic), calibration-in-the-large (CITL), calibration slope, observed-to-expected (O/E) ratio, calibration plots, and decision curve analysis (DCA). A logistic recalibration (intercept and slope) using the original linear predictor was then fitted on the full cohort and applied uniformly to subgroups (sex; mental health, learning disability). Among 32,410 adults (fall rate 2.07%), the model showed good discrimination (C-statistic = 0.777). Before recalibration, calibration was suboptimal (CITL = 1.36; O/E = 1.60; slope = 1.22). Recalibration restored alignment (CITL *≈* 0; O/E *≈* 1; slope *≈* 1) without changing discrimination. Subgroup analyses revealed degraded performance in learning disability groups (e.g., AUC 0.696–0.739; marked underprediction), whereas sex and mental-health-only groups were closer to overall performance. DCA indicated positive net benefit across clinically relevant thresholds (10–25%). The *eFalls* model showed reasonable performance in working-age adults receiving mental health or learning disability services following simple recalibration. However, discrimination was lower among individuals with learning disabilities, suggesting that recalibration alone may be insufficient and that further model refinement and validation in this subgroup are warranted.

**Supplementary Information:**

The online version contains supplementary material available at https://doi.org/10.1038/s41598-026-51298-0.

## Introduction

Falls are a leading cause of morbidity, disability, and healthcare utilization across all age groups, placing a substantial burden on individuals and healthcare systems worldwide^1,2^. Although most fall risk prediction tools have focused on older adults–where risk factors such as frailty, multimorbidity, and medication count are prevalent–emerging evidence indicates that younger individuals, particularly those with mental health conditions or learning disabilities (MH/LD), are also at elevated risk^3–5^. This increased risk may stem from a range of factors including psychotropic medication use, cognitive impairments, reduced mobility, and social or environmental vulnerabilities^6,7^.

Several fall risk models have been developed for use in inpatient and acute settings. Tools such as STRATIFY^8^, the Hendrich II Fall Risk Model^9^, and the Morse Fall Scale^10^ are widely used but often suffer from poor calibration, limited external validity, and a lack of generalizability beyond their development cohorts. These tools also typically rely on clinician-reported checklists and do not leverage the growing availability of longitudinal electronic health record (EHR) data. In contrast, the emergence of large-scale EHR systems has facilitated the development of data-driven fall prediction models that utilize routinely coded clinical information. Recent machine learning approaches, including random forest and deep learning-based models^11,12^, have shown promising discrimination performance. However, they are frequently limited by issues of transparency, reproducibility, and practical implementation in real-world clinical workflows^13,14^.

A notable contribution in this space is the *eFalls* model^15^, using the Secure Anonymised Information Linkage (SAIL) Databank, which contains longitudinal, routinely collected, anonymised EHR data sources from around 5 million people across Wales, with linked primary care, emergency department attendance, hospital admissions and Office for National Statistics mortality data from adults aged *≥*65 years. The model employs a LASSO-regularised logistic regression framework to predict 12-month risk of fall-related emergency hospital attendance. Predictor variables include demographic features, lifestyle factors (e.g., smoking, alcohol), and over 50 clinical conditions derived from the electronic Frailty Index (eFI)^16^, offering a transparent and scalable approach.

The *eFalls* model demonstrated strong discrimination in external validation (C-statistic = 0.82) and performed reasonably well across subgroups stratified by frailty, sex, and socioeconomic deprivation. However, calibration performance was more variable–particularly among underweight and severely frail individuals–with a negative calibration-in-the-large (CITL), indicating systematic overprediction. To address this, the authors applied a recalibration procedure using a logistic regression model on the original linear predictor, updating the intercept and slope to improve absolute risk estimation^15^. Despite its strengths, the *eFalls* model was developed exclusively in older adults and its performance in younger populations remains unknown. Many of the predictors it uses–such as medication count, comorbidity burden, and frailty indicators–are also relevant to working-age adults with MH/LD conditions^4,6^. Yet, there has been little empirical investigation into whether *eFalls* or similar models generalise to such populations.

A large integrated teaching NHS foundation trust in the north of England provides a unique setting to explore this question. The Trust provides a wide range of specialist services to support recovery and meet local needs, including services for individuals with diverse mental health needs and learning disabilities. Although not all individuals in the dataset have a formal MH/LD diagnosis, this cohort provides an opportunity to assess whether a tool like *eFalls* can be meaningfully applied or adapted to mental health settings. Specific challenges include the prevalence of psychotropic medication use, behavioural dysregulation, and atypical risk profiles, which may differ from those seen in older adults.

In this study, we aim to externally validate the *eFalls* model using clinical data from working-age adults (18–65 years). We evaluate the model’s discrimination, calibration, and clinical utility, and explore the use of recalibration techniques to improve its performance. We further investigate performance in subgroups using the status of sex, mental health and learning disability. This work addresses a critical evidence gap and contributes to the broader effort to improve the fairness, robustness, and applicability of fall risk models across diverse NHS populations.

## Methods

### Data source and study population

This study constitutes a retrospective external validation of the *eFalls* prediction model using routinely collected electronic health records (EHRs) from a a large integrated teaching national health service (NHS) foundation trust (hereafter referred to as the “Trust”) at Wakefield, England. The aim was to assess the performance of the *eFalls* model in a working-age adult population (18–65 years), diverging from the original model’s focus on adults aged 65 and older^15^.

Patient inclusion was restricted to those under active care by Trust who were between 18 and 65 years of age as of February 2023. The baseline index date was defined as the most recent hospital contact occurring on or before February 28, 2023. For each included patient, their NHS number was used to link General Practice (GP) records, provided there was an existing data-sharing agreement between Trust and the relevant GP practice. Patients whose GP practices did not have such agreements, or who had opted out of research data sharing, were excluded from the study.

Data were collected from both Trust and GP records to ensure a comprehensive view of each patient’s clinical history. This included retrospective data (prior to the baseline) to derive predictors and 12 months of prospective data (after the baseline) to ascertain the outcome. After data linkage and cleaning, the dataset was split into an individualised historical (“past”) and follow-up (“future”) set for each patient. All predictor variables were derived from the historical dataset, while outcome labels were assigned based on future events.

To ensure fidelity to the original model, the clinical code mapping for all variables strictly followed the specifications provided in the original *eFalls* study. The coding systems (CTV3, Read v2, SNOMED CT) were harmonised, and a validation process was performed to verify that no relevant codes were lost or misclassified during merging. Only the exact clinical codes (not parent or child concepts) specified in the original study were used to define each variable.

All 75 predictors included in the published eFalls model were available within the Trust-linked dataset through harmonised CTV3, Read v2, and SNOMED CT coding systems. No predictors required proxy substitution. Clinical code mapping strictly followed the original study specification to ensure structural equivalence between development and validation models.

The primary outcome was defined as any emergency department (ED) attendance or hospitalisation for falls or fractures occurring within 12 months following the baseline date. This outcome aligns with that used in the original *eFalls* study and was derived using diagnostic codes indicating fall-related injuries. The original *eFalls* model includes 75 predictors selected using LASSO-penalised logistic regression, encompassing demographic characteristics (e.g., age, sex), body mass index (BMI), lifestyle factors (e.g., smoking status, alcohol consumption), medication count (referred to as polypharmacy in the original eFalls paper), and a wide array of diagnostic indicators based on the electronic Frailty Index and additional morbidity markers.

**Ethics approval and data governance.** All methods were carried out in accordance with relevant guidelines and regulations. The study was conducted in accordance with the Declaration of Helsinki and approved by the Health Research Authority (HRA) and Health and Care Research Wales (HCRW) (Reference: 24/PR/1367; approved 17 December 2024). Informed consent was not required for this pseudo-anonymised data study. A collaboration agreement for this project was established between University of Southampton, University of Huddersfield and South West Yorkshire Partnership Teaching NHS Foundation Trust. In addition, a Privacy Impact Assessment (PIA) was completed and approved by the University of Huddersfield to ensure that the use of anonymised data complied with relevant data protection legislation.

### Handling of missing data

Missingness in the Trust dataset primarily affected categorical lifestyle variables, most notably body mass index (BMI) (9.8%) and alcohol consumption (77.0%). To preserve fidelity with the original eFalls model specification, missing values for these variables were treated as explicit “Missing” categories rather than being imputed or excluded. This approach mirrors the development and external validation strategy described in the original eFalls study, where missingness was incorporated as an informative category within the model.

Age, medication count, and smoking status were fully observed in the validation dataset. For diagnostic indicators derived from electronic health records (e.g., comorbid conditions), the absence of a recorded diagnostic code was interpreted as absence of the condition, consistent with standard practice in routinely coded primary care and hospital datasets and aligned with the original model derivation.

No additional imputation procedures were performed. This strategy ensured direct transportability of the published regression coefficients and avoided introducing modelling assumptions inconsistent with the original eFalls framework.

### Subgroup analyses: sex, mental health, and learning disability

Subgroup analyses were conducted to assess variation in model discrimination and calibration across clinically relevant strata. Analyses were stratified by sex(male/female), mental health status (MH), and learning disability status (LD).

MH status was defined using a binary indicator (MH_stratifier), set to positive if an individual had at least one of the following conditions: depression, general_mental_health, self_harm, severe_mental_illness, or stress. These variables are part of the original *eFalls* predictor set; therefore, MH-based subgroup analyses are descriptive and non-causal.

LD status was defined using a separate binary indicator derived from structured diagnostic data. LD was not included among the original *eFalls* predictors, allowing its use as an external validation stratifier.

To examine overlapping vulnerability, participants were additionally stratified by the joint presence or absence of MH and LD. Notation such as MH_only, LD_only, MH_and_LD, and No_MH_No_LD denotes mutually exclusive groups defined by combinations of MH and LD status, with complementary groupings (e.g., Any_MH, No_LD) used for marginal comparisons. This enabled assessment of model behaviour across increasingly complex clinical profiles. While some subgroups were small and require cautious interpretation, these analyses provide insight into the generalisability and equity of fall-risk prediction across heterogeneous populations.

### Evaluation metrics

Model performance in the external working-age cohort and across subgroups was assessed using measures of discrimination and calibration, capturing risk ranking ability and agreement between predicted and observed outcomes. The full set of published *eFalls* coefficients^15^ was applied unchanged to the Trust dataset to generate predicted risks.

**Discrimination.** Discrimination was evaluated using the C-statistic (area under the ROC curve), which quantifies the model’s ability to distinguish individuals who experienced a fall from those who did not. Values range from 0.5 (no discrimination) to 1.0 (perfect discrimination).

**Calibration.** Calibration was assessed using complementary metrics: 1) Calibration-in-the-large (CITL), measuring average under- or over-prediction (0 indicates perfect agreement); 2) Calibration slope, assessing agreement between predicted risk spread and observed outcome variability (ideal value = 1); 3) Observed-to-Expected (O/E) ratio, comparing total observed to predicted events (ideal value = 1). Calibration plots were also generated by grouping individuals into deciles of predicted risk and comparing mean predicted probabilities with observed event rates; deviations from the identity line indicate miscalibration across the risk spectrum.

**Clinical Utility.** Clinical utility was evaluated using decision curve analysis (DCA), which estimates net benefit relative to treat-all and treat-none strategies. Consistent with the original *eFalls* study, analyses focused on risk thresholds between 10% and 25%, identified a priori as clinically actionable for fall-prevention decision-making.

### Application of the original *eFalls* prediction model

The *eFalls* risk score was originally developed using penalised logistic regression (LASSO) applied to routinely collected electronic health record data^15^. The model estimates the probability of a fall event using a linear predictor composed of demographic, lifestyle, and clinical variables.

The predicted probability of falling is calculated using the logistic transformation: $P(\text {fall}) = \frac{1}{1 + \exp (-LP)}$, where the linear predictor *LP* is defined as $LP = \beta _0 + \sum _{j=1}^{p} \beta _j X_j$. Here, $\beta _0$ denotes the model intercept, $X_j$ represents the predictor variables, and $\beta _j$ are the corresponding regression coefficients estimated in the original model.

For the present validation study, the linear predictor for each individual was calculated using the published coefficients from the original *eFalls* model without modification^15^. The complete list of predictors and their corresponding regression coefficients from the final penalised model are provided in Supplementary Table S1 to ensure transparency and reproducibility.

### Model recalibration

To account for differences in baseline risk between the original *eFalls* development population and the external Trust cohort, model recalibration was performed. Following the strategy used in the original *eFalls* external validation, a simple intercept-and-slope recalibration was applied.

Recalibration was implemented by fitting a logistic regression model in the external data with the original *eFalls* linear predictor as the sole covariate: $\text {logit}(Y) = \alpha + \beta \,LP_{\textit{eFalls}}$, where *α* adjusts for differences in outcome incidence and *β* corrects for global over- or underfitting. This procedure updates calibration while preserving the original ranking of predicted risks.

Recalibration was performed once using the full working-age cohort, and the adjusted model was applied uniformly across all subgroups to avoid overfitting and to ensure comparability of subgroup performance estimates.

## Sample characteristics

A total of 32410 individuals aged 18–65 were included in the validation cohort. Table 1 presents a comprehensive summary of the demographic, clinical, and behavioral characteristics of the study population, stratified by future fall status. For each categorical variable, both the total counts and the distribution of falls (Fall = 1) versus no falls (Fall = 0) are shown, along with percentage breakdowns. Two continuous variables, *Age* and *Medication count*, are also included, summarized using mean, median, and interquartile range (IQR).

Table 2 presents a comprehensive summary of the prevalence of a wide array of medical conditions recorded in the study population, alongside the proportion of individuals with each condition who subsequently experienced a future fall. The table is structured to report the total number and percentage of individuals active for each condition, followed by the number and percentage who experienced a fall (*Fall*), and those who did not (*No Fall*). This breakdown allows for a clearer understanding of both condition-specific burden and relative fall risk within each subgroup. It is worth noting that the medical conditions listed are exactly the same predictors used in the original eFalls model.

## Validation results and discussions

This section presents a comprehensive evaluation of the eFalls model on external data from Trust. We begin by assessing the model’s overall performance using bootstrap resampling to estimate confidence intervals for key metrics at the population level. Next, we focus on direct application of the model to the full Trust cohort to examine point estimates and explore calibration and discrimination in detail through a series of visualisations and summary statistics. Finally, we analyse model performance across key stratified subgroups to assess whether prediction quality varies across different patient characteristics.

**Table 1:** Descriptive statistics of demographic and clinical features stratified by future fall status. Percentages in the Fall and No Fall columns are calculated within outcome groups.

Category	Group	Total		Fall		No Fall	
		N	(%)	N	(%)	N	(%)
Sex	Female	16946	52.3	401	59.9	16545	52.1
	Male	15464	47.7	269	40.1	15195	47.9
BMI Category	Underweight	2237	6.9	38	5.7	2199	6.9
	Normal	9871	30.5	169	25.2	9702	30.6
	Obese	9611	29.7	304	45.4	9307	29.3
	Missing	3184	9.8	11	1.6	3173	10.0
	Overweight	7507	23.2	148	22.1	7359	23.2
Smoking	Current	13042	40.2	229	34.2	12813	40.4
	Ex/Never	19368	59.8	441	65.8	18927	59.6
Alcohol	Harmful	3447	10.6	73	10.9	3374	10.6
	Higher Risk	35	0.1	1	0.1	34	0.1
	Prev. Higher Risk Harmful	101	0.3	6	0.9	95	0.3
	Zero Alcohol	2380	7.3	86	12.8	2294	7.2
	Missing	24966	77.0	478	71.3	24488	77.2
	Lower Risk	1481	4.6	26	3.9	1455	4.6
Overall Mental Health	Yes	11132	34.3	277	41.3	10855	34.2
	No	21278	65.7	393	58.7	20885	65.8
Learning Disability	Yes	1771	5.5	163	24.3	1608	5.1
	No	30639	94.5	507	75.7	30132	94.9
Age	Mean, Median [Q1–Q3]	37.0, 35.0 [27.0–47.0]					
	Fall group	46.4, 50.0 [37.0–57.0]					
	No Fall group	36.8, 35.0 [27.0–47.0]					
Medication count	Mean, Median [Q1–Q3]	3.2, 0.0 [0.0–5.0]					
	Fall group	8.6, 8.0 [0.0–14.0]					
	No Fall group	3.0, 0.0 [0.0–5.0]

**Table 2:** Prevalence of medical conditions and associated fall rates. Total (%) is calculated over the full cohort (N=32,410). Fall (%) and No Fall (%) are calculated within outcome groups (Fall: n=670; No Fall: n=31,740).

Medical Condition	Total	Total (%)	Fall	Fall (%)	No Fall	No Fall (%)
Abdominal Pain	5879	18.1	161	24.0	5718	18.0
Activity Limitation	1020	3.1	49	7.3	971	3.1
Anaemia	3459	10.7	144	21.5	3315	10.4
Asthma	7596	23.4	200	29.9	7396	23.3
Atrial Fibrillation	149	0.5	9	1.3	140	0.4
Back Pain	11921	36.8	342	51.0	11579	36.5
Bone Disease	0	0.0	0	0.0	0	0.0
Cancer	299	0.9	8	1.2	291	0.9
Cognitive Impairment	46	0.1	6	0.9	40	0.1
Copd	678	2.1	49	7.3	629	2.0
Dementia	221	0.7	25	3.7	196	0.6
Depression	5107	15.8	121	18.1	4986	15.7
Diabetes	2300	7.1	144	21.5	2156	6.8
Dizziness	3266	10.1	131	19.6	3135	9.9
Dressing Grooming Problems	694	2.1	101	15.1	593	1.9
Faecal Incontinence	396	1.2	60	9.0	336	1.1
Falls	2310	7.1	210	31.3	2100	6.6
Fatigue	1589	4.9	55	8.2	1534	4.8
Foot Problems	331	1.0	22	3.3	309	1.0
Fracture	43	0.1	1	0.1	42	0.1
Fragility Fracture	404	1.2	18	2.7	386	1.2
General Mental Health	5958	18.4	157	23.4	5801	18.3
Headache	8082	24.9	217	32.4	7865	24.8
Hearing Impairment	2725	8.4	97	14.5	2628	8.3
Heart Failure	168	0.5	17	2.5	151	0.5
Housebound	5299	16.3	245	36.6	5054	15.9
Hypertension	12639	39.0	449	67.0	12190	38.4
Hypotension Or Syncope	13052	40.3	455	67.9	12597	39.7
Inflammatory Arthritis	1009	3.1	58	8.7	951	3.0
Inflammatory Bowel Disease	127	0.4	7	1.0	120	0.4
Liver Problems	191	0.6	6	0.9	185	0.6
Meal Preparation Problems	341	1.1	55	8.2	286	0.9
Medication Management	402	1.2	67	10.0	335	1.1
Memory Concerns	78	0.2	12	1.8	66	0.2
Mobility Problems	1110	3.4	157	23.4	953	3.0
Mono Or Hemiparesis	217	0.7	19	2.8	198	0.6
Motor Neuron Disease	0	0.0	0	0.0	0	0.0
Musculoskeletal Problems	13051	40.3	388	57.9	12663	39.9
Osteoarthritis	1921	5.9	127	19.0	1794	5.7
Osteoporosis	414	1.3	33	4.9	381	1.2
Palliative Care	52	0.2	7	1.0	45	0.1
Parkinsonism And Tremor	80	0.2	11	1.6	69	0.2
Peptic Ulcer Disease	411	1.3	15	2.2	396	1.2
Peripheral Neuropathy	726	2.2	51	7.6	675	2.1
Peripheral Vascular Disease	487	1.5	47	7.0	440	1.4
Requirement For Care	3242	10.0	261	39.0	2981	9.4
Respiratory Disease	1108	3.4	56	8.4	1052	3.3
Seizures	1542	4.8	134	20.0	1408	4.4
Self Harm	2089	6.4	39	5.8	2050	6.5
Severe Mental Illness	7784	24.0	213	31.8	7571	23.9
Skin Ulcer	697	2.2	76	11.3	621	2.0
Sleep Problems	3537	10.9	129	19.3	3408	10.7
Social Vulnerability	5899	18.2	282	42.1	5617	17.7
Stress	793	2.4	31	4.6	762	2.4
Stroke	572	1.8	63	9.4	509	1.6
Thyroid Problems	3514	10.8	148	22.1	3366	10.6
Urinary Incontinence	1416	4.4	127	19.0	1289	4.1
Urinary System Disease	8087	25.0	263	39.3	7824	24.7
Visual Impairment	3402	10.5	191	28.5	3211	10.1
Washing Bathing Problems	201	0.6	20	3.0	181	0.6
Weakness	184	0.6	17	2.5	167	0.5
Weight Loss	2791	8.6	114	17.0	2677	8.4

**Table 3:** Comparison of model performance metrics between the current external validation on Trust data and the published eFalls external validation results on the Connected Bradford dataset.

	Metric	Trust Original	Trust Recalibrated	eFalls Original	eFalls Recalibrated
Calibration Slope	Point	1.222	1.000	1.248	1.000
	95% CI	[1.139, 1.312]	[0.932, 1.073]	[1.244, 1.265]	[1.000, 1.013]
CITL	Point	1.357	−0.001	−0.931	0.000
	95% CI	[1.032, 1.696]	[−0.234, 0.240]	[−0.938, −0.920]	[−0.009, 0.010]
O/E Ratio	Point	1.604	1.000	0.432	1.000
	95% CI	[1.584, 1.623]	[0.984, 1.016]	[0.430, 0.437]	[0.992, 1.009]
C-statistic (AUC)	Point	0.777	0.777	0.825	0.825
	95% CI	[0.757, 0.795]	[0.757, 0.795]	[0.824, 0.828]	[0.825, 0.828]

### Bootstrap-based performance estimation

To quantify uncertainty in model performance metrics, we applied bootstrap resampling 1,000 times to the external validation dataset, maintaining the original class distribution via stratification. This enabled the estimation of 95% confidence intervals (CI) for calibration and discrimination metrics, thus allowing a more nuanced interpretation beyond point estimates alone. The results for both the original and recalibrated models are summarised in Table 3.

**Before Recalibration.** The external validation revealed clear evidence of systematic miscalibration when the original model was applied to the Trust population. The calibration-in-the-large (CITL) was estimated at 1.36 (95% CI: 1.03–1.70), indicating that, on average, the model significantly underestimated the absolute risk of falls. A positive CITL value reflects a situation where the observed event rate is substantially higher than what the model predicts. This is further corroborated by the observed-to-expected (O/E) ratio of 1.60 (95% CI: 1.58–1.62), which quantifies the degree of underestimation by showing that the number of observed fall events was roughly 60% higher than expected under the model.

In addition to the intercept shift, the calibration slope was 1.22 (95% CI: 1.14–1.31), suggesting that predicted probabilities were under-dispersed. That is, the model did not sufficiently spread out the risk estimates, leading to compression of predicted values toward the mean. This under-dispersion manifests in underprediction for high-risk individuals and overprediction for those at lower risk, and is commonly observed when a model is trained on one population but applied to another with a different risk structure or predictor-outcome relationships.

Despite these calibration issues, the model maintained good discrimination ability, as reflected in a C-statistic (AUC) of 0.78 (95% CI: 0.76–0.80). This indicates that the model was effective at ranking individuals by their relative risk of falling, even if the predicted probabilities themselves were systematically misaligned with the actual outcome rates.

**After Recalibration.** To correct the observed miscalibration while preserving discrimination, a simple logistic recalibration was applied, as applied by the original efall research^15^. This involved fitting a logistic regression of the binary outcome on the logit-transformed predicted probabilities, effectively adjusting the slope and intercept of the model. After recalibration, the CITL was brought close to zero (−0.001; 95% CI: −0.234–0.240), indicating that the recalibrated model no longer systematically over- or under-predicted the average risk. The O/E ratio similarly converged to 1.00 (95% CI: 0.98–1.02), consistent with a model whose predicted risks now align with observed event frequencies.

The recalibration slope was 1.00 (95% CI: 0.93–1.07), confirming that the dispersion of predicted probabilities matched that of the observed outcomes post-adjustment. This value close to unity suggests that the relative differences in risk across patients are now appropriately scaled. Importantly, the way how the model is recalibrated by does not change the rank of risks, hence results in exact same discriminatory capacity, the recalibrated model retained its discriminatory capacity, with a C-statistic remaining at 0.78 (95% CI: 0.76–0.80).

**Comparison with Published eFalls External Validation.** To further assess the relative external validity of the eFalls model, we compared its performance on the Trust cohort with results reported in the original eFalls external validation, which used data from the Connected Bradford dataset^17^. That validation included 81,685 individuals across 76 practices, with a fall event rate of 2.9%, while our Trust validation cohort comprised 32,410 individuals with a slightly lower event rate of 2.07%.

In terms of discrimination, the eFalls model achieved a higher C-statistic in its original external validation (0.825, 95% CI: 0.824–0.828) than in our setting (0.777, 95% CI: 0.757–0.795), suggesting a modest decline in the model’s ability to distinguish fallers from non-fallers in the Trust population. This may be due to differences in local population characteristics, clinical coding practices, or data completeness.

For calibration, both validations revealed systematic miscalibration prior to recalibration but in opposite directions. In the Connected Bradford data, the model overpredicted risk (CITL = −0.931; O/E = 0.432), while in Trust it underpredicted risk (CITL = 1.357; O/E = 1.604). This divergence likely reflects differences in baseline event rates and case mix. Nonetheless, in both settings, simple logistic recalibration was effective: CITL values approached zero, and O/E ratios converged to 1.00, indicating that recalibration successfully realigned average predicted risk with observed outcomes.

The calibration slope was slightly above 1.0 in both pre-recalibrated validations (1.248 and 1.222, respectively), indicating under-dispersion and suggesting that predicted probabilities were too concentrated around the mean. After recalibration, both slopes corrected to approximately 1.000, demonstrating improved alignment between predicted and actual risk gradients.

Taken together, the results confirm that while the eFalls model retains strong discriminatory ability across external populations, its calibration can vary substantially depending on the underlying event prevalence and data characteristics. In our Trust validation, the model significantly underpredicted risk prior to recalibration, contrasting with overprediction observed in the original eFalls external validation. Despite these differences, logistic recalibration consistently corrected systematic misalignment in both settings–bringing calibration slope, CITL, and O/E ratios close to ideal values without compromising discrimination. These findings underscore the importance of validating and recalibrating clinical prediction models prior to local deployment. When combined with clinical input on relevant decision thresholds, recalibrated models like eFalls can support effective, context-specific risk stratification strategies in routine care.

**On the Direction of Calibration-in-the-Large.** Interestingly, although the Trust validation dataset has a lower event rate (2.07%) than the original eFalls development cohort (4.9%), we observed a positive calibration-in-the-large (CITL = 1.36) — indicating underprediction of risk. This might appear counterintuitive, as one would typically expect a model trained on a higher-prevalence population to overpredict risk when applied to a lower-prevalence cohort, as was indeed observed in the original eFalls validation on the Connected Bradford dataset (CITL = −0.93). However, this inversion might suggest that the relative importance of specific predictors in the original eFalls model–trained predominantly on individuals aged 65 and above–does not fully align with risk factor patterns in our validation cohort, which includes a younger working-age population. The characteristics of fall risk in younger adults may differ substantially, and certain features that were strongly predictive in the older population may carry less weight in the Trust setting. Conversely, other factors not prioritised in the original model may play a greater role. As a result, the model may systematically underpredict fall risk in Trust, even though the overall prevalence is lower, because it may fail to adequately capture the risk signal in this distinct population.

This highlights the complexity of model transportability, particularly across populations with different age profiles, health behaviours, or risk factor distributions. Nonetheless, the overall external validation performance of the eFalls model on Trust data remains reasonably strong. The model achieved a C-statistic of 0.777, indicating good discriminatory ability, and after recalibration, both the calibration slope (1.000) and calibration-in-the-large (–0.001) suggest excellent alignment between predicted and observed risks. This supports the model’s continued applicability, provided local recalibration is performed to account for population-specific differences.

### Point estimates and calibration diagnostics

We applied the original eFalls model directly to the external Trust validation data to obtain point estimates of key performance metrics. Without any bootstrapping, the original model exhibited a calibration slope of 1.223, indicating moderate under-dispersion in predicted risks–i.e., predicted probabilities were compressed toward the middle of the risk spectrum. The calibration-in-the-large (CITL) was 1.358, reflecting substantial underestimation of the average event rate, consistent with the observed-to-expected (O/E) ratio of 1.604. This means that the total number of observed falls was approximately 60% higher than the model predicted. The model retained good discrimination, with an area under the ROC curve (AUC) of 0.777, suggesting reasonable overall accuracy in probability estimation.

After recalibration via logistic regression on the logit-transformed predicted probabilities, the recalibrated model achieved near-ideal calibration characteristics: the calibration slope was corrected to 1.000 and the CITL to −0.000, resulting in an O/E ratio of 1.000. These adjustments realigned the predicted probabilities with the observed event distribution in the external population, while preserving the original AUC (0.777).

**Fig. 1:**
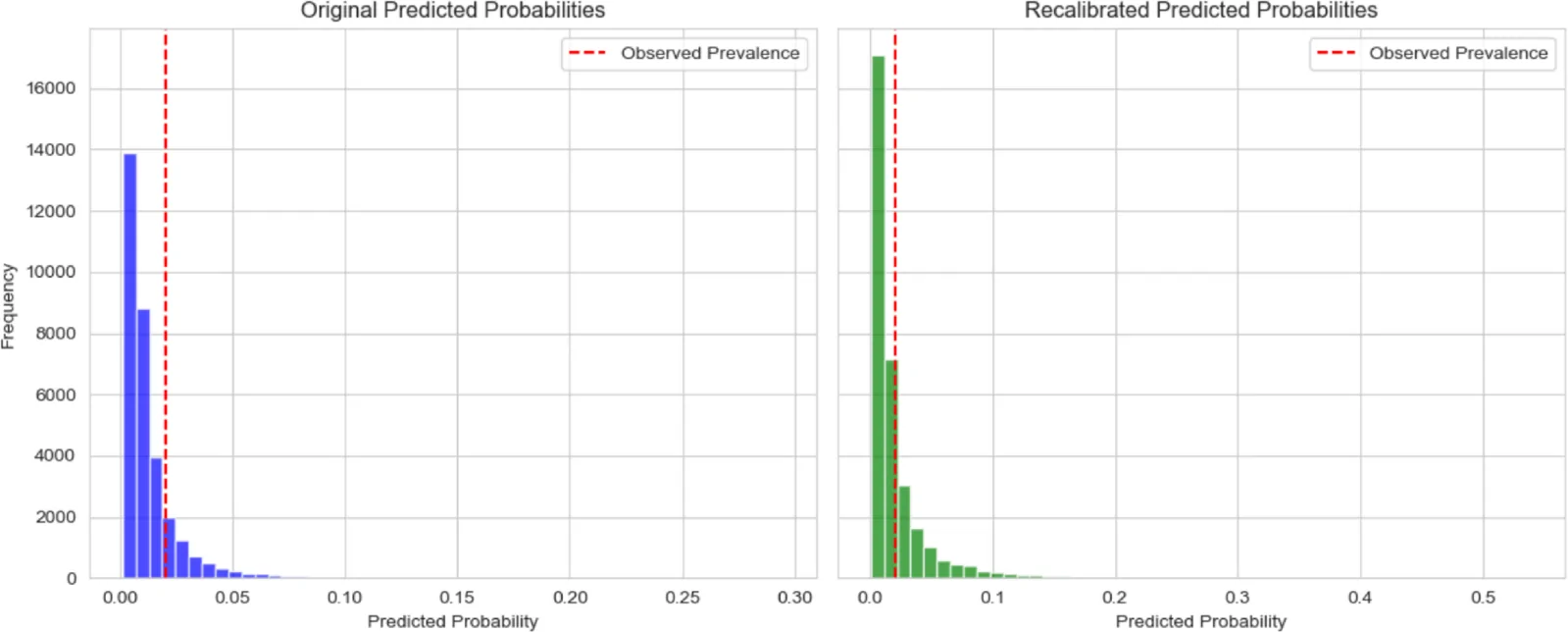
Distribution of risks before and after recalibration.

To visualise the model behaviour, we plotted histograms of predicted probabilities for both the original and recalibrated models (Figure 1). For the original model, the predicted risk distribution was skewed toward lower probabilities, with very few patients assigned a risk above 10%,, indicating the model’s tendency to conservatively assign low-risk estimates to most individuals. The red vertical line indicates the observed prevalence of falls (2.07%), which lies noticeably to the right of the predicted probability peak, again underscoring the model’s underestimation of risk.

Moreover, the distribution shows a lack of extreme predictions (e.g., probabilities >0.30), suggesting that the model does not sufficiently differentiate between low- and high-risk individuals. This observation aligns with the calibration slope exceeding 1.0, indicating an under-dispersion of predicted risks. These suggest that while the model ranks individuals well in terms of relative risk, it does not confidently identify the tails of the risk spectrum in the new population. In contrast, the recalibrated model yielded a slight rightward shift in the probability distribution, aligning more closely with the empirical event rate and improving calibration without distorting the underlying ranking of individuals by risk.

**Calibration Curves with Grouped Estimates and LOWESS Smoothing.**

**Fig. 2:**
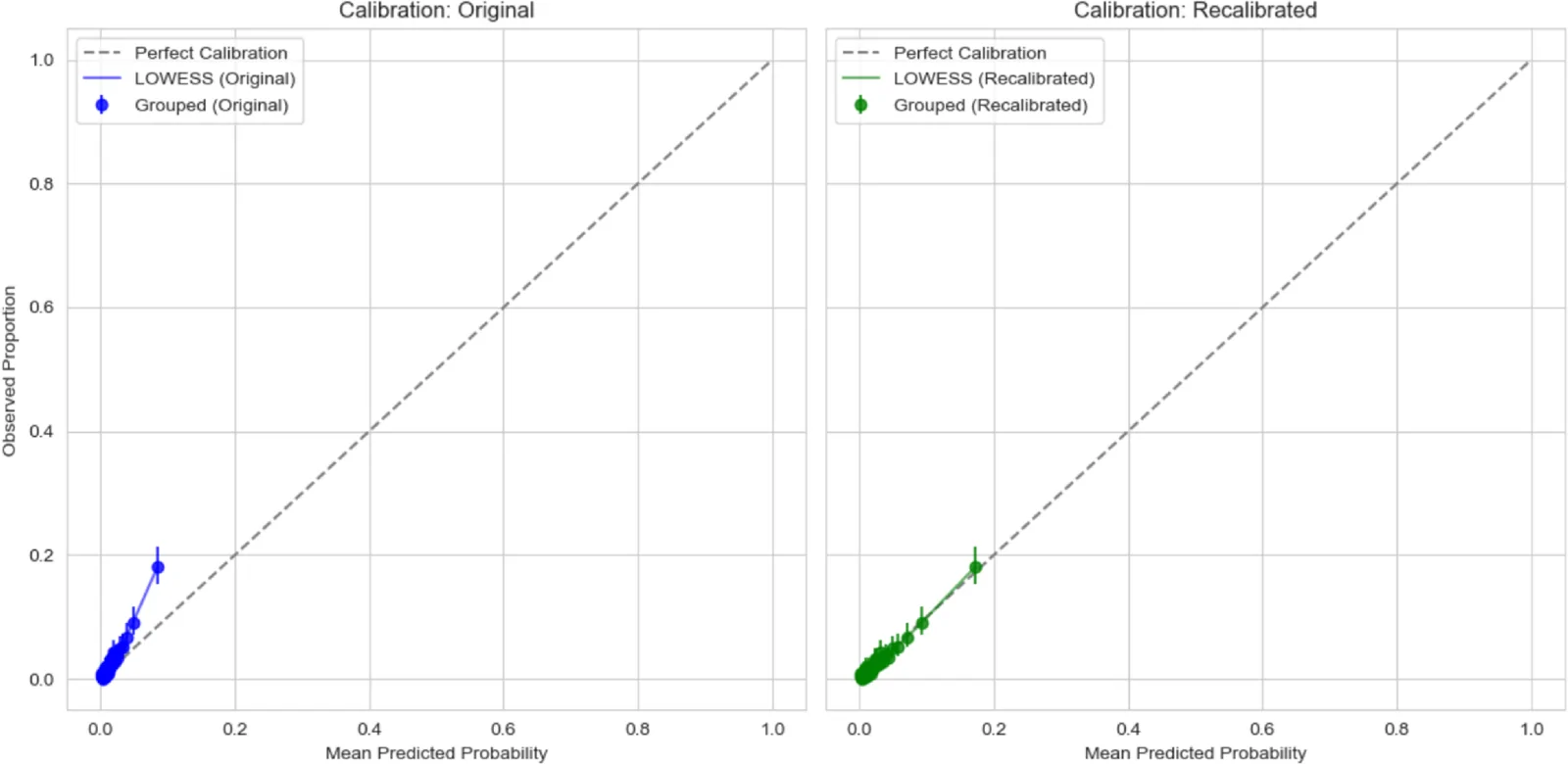
Calibration plots for before and after recalibration.

To further assess how well the predicted probabilities aligned with observed event rates across the full range of risk, we constructed calibration plots for both the original and recalibrated models, as shown in Figure 2. Patients were first grouped into 50 equal-sized bins based on their predicted probabilities using quantile-based binning, ensuring that each bin contained a similar number of individuals. For each bin, we computed the average predicted probability (horizontal axis) and the corresponding observed fall rate (vertical axis), along with 95% confidence intervals for the observed proportions using Wilson’s method. These grouped statistics were visualised using error bars, providing a non-parametric, interpretable summary of local calibration.

In the original model, the grouped calibration plot revealed systematic misalignment between predicted and observed risks across most of the probability spectrum. The plotted points and confidence intervals consistently lay above the 45-degree reference line, especially in the mid- to higher-risk range, indicating that the model persistently underpredicted absolute risk levels. The accompanying LOWESS curve further confirmed this deviation, tracing a trajectory that remained above the identity line throughout and suggesting consistent underestimation. These findings are consistent with earlier summary metrics such as the elevated calibration-in-the-large (CITL) and the high observed-to-expected (O/E) ratio.

In contrast, the recalibrated model showed near-ideal alignment with the reference line. The grouped points closely followed the 45-degree line across the entire risk spectrum, and the LOWESS-smoothed curve overlapped it almost completely, with only minimal deviations. The confidence intervals around the observed event rates also became narrower and more symmetrical, indicating both improved point-wise calibration and reduced local uncertainty. Taken together, these plots illustrate that the recalibration procedure not only corrected the global offset in predicted probabilities but also restored local fidelity between predicted and observed risk across different strata of patients.

The inclusion of both grouped calibration points and LOWESS smoothing allowed us to diagnose subtle patterns of miscalibration that may be missed by single-number summaries. While the original model demonstrated good discrimination, the plots made explicit its tendency to systematically underestimate risk–especially among higher-risk individuals. After recalibration, the improved alignment enhances the clinical utility of the model by enabling more trustworthy probabilistic predictions for decision support applications.

**Decision Curve Analysis.**

To evaluate the clinical utility of the original and recalibrated models, we conducted a decision curve analysis (DCA), which quantifies the net benefit of using model-based predictions to guide interventions across a range of decision thresholds. For each threshold probability *t ∈ [0.01, 0.49]*, we computed the net benefit of treating individuals whose predicted fall risk exceeded *t*. Net benefit is defined as the true positive rate minus a penalty for false positives, scaled by the odds of the threshold $\frac{t}{1 - t}$, and represents the expected value of a decision rule in terms of correctly identifying patients at risk while avoiding unnecessary interventions.

**Fig. 3:**
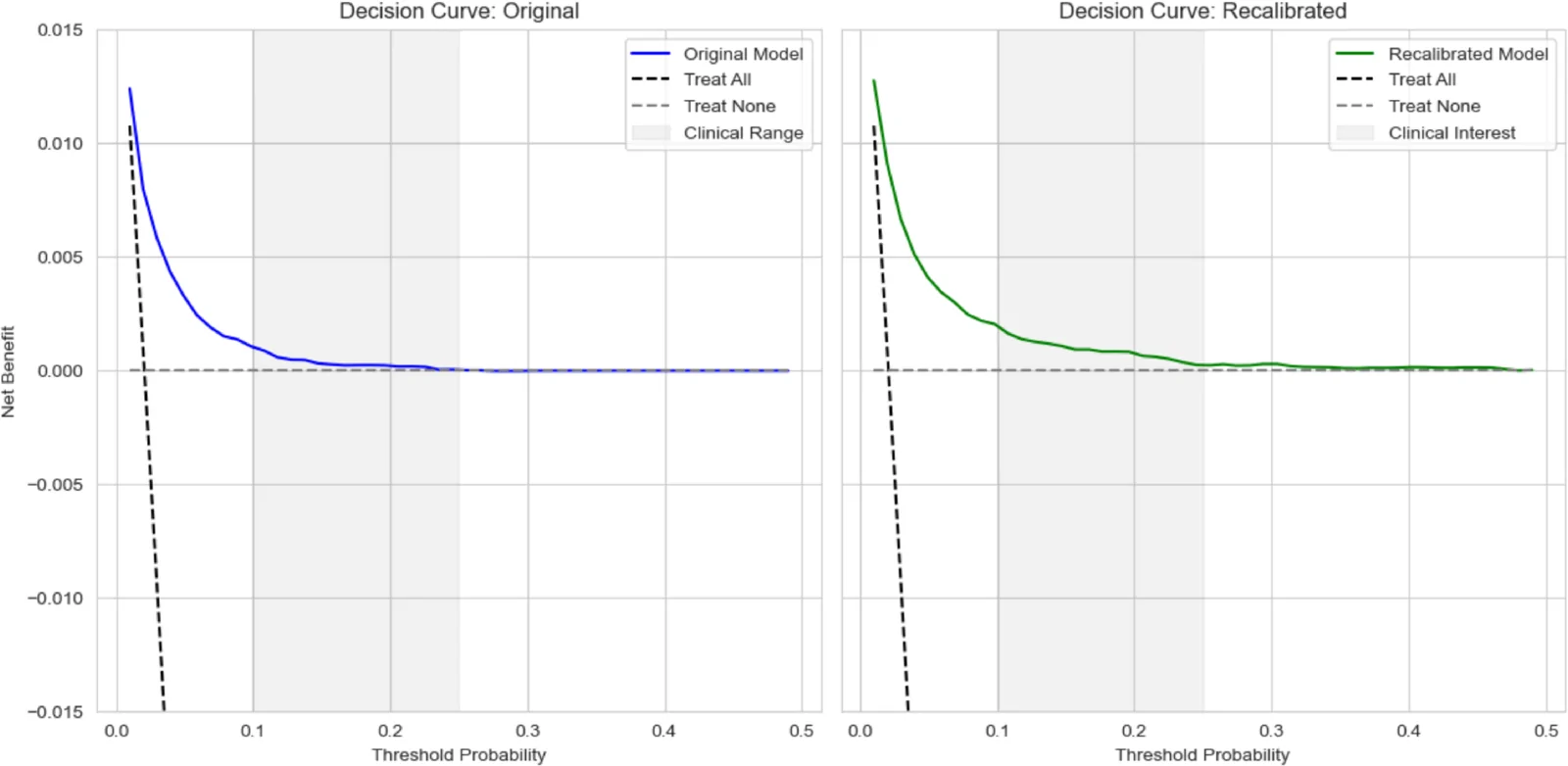
Decision curve analysis before and after recalibration.

Figure 3 displays the DCA plots for both the original and recalibrated models, with vertical shading highlighting the clinically relevant range of thresholds (10% to 25%) consistent with what original eFall paper specifies. For both models, the decision curves lie above the “treat none” and “treat all” strategies within this range, indicating that risk-guided intervention would offer greater clinical value than either extreme strategy. However, the recalibrated model consistently yields a slightly higher net benefit across most thresholds, particularly within the 10–25% window. This suggests that recalibration not only improves probabilistic calibration but also enhances downstream clinical decision-making performance.

It is worth noting that the absolute values of net benefit are small (in the range of ±0.015), reflecting the low event prevalence (2.07%) in the validation cohort. Accordingly, we zoomed the y-axis around zero to highlight these differences. The recalibrated model shows more stable and marginally higher gains, implying that its improved alignment with observed risks translates into a better balance between sensitivity and specificity when used for guiding fall prevention strategies.

It’s worth emphasizing that While observed net benefit may appear modest, for instance, the net benefit being around 0.002 in the case of 0.1 threshold probability, it is important to consider the context of a low-prevalence outcome. Given that the prevalence of falls in the Trust validation dataset is 2.07%, this corresponds to approximately 21 fallers per 1,000 patients. A net benefit of 0.002 implies that, compared to a strategy of treating no one, the model enables two additional true-positive identifications per 1,000 patients without increasing the number of unnecessary interventions. In other words, the model provides a marginal gain in identifying fall-risk individuals beyond random allocation or blanket treatment strategies. This also reflects the challenge of improving decision-making in low-risk populations, where the absolute number of events is small and the scope for gain is inherently constrained. Nonetheless, even modest net benefits can be clinically meaningful when scaled across large populations or when interventions are costly or carry potential harms.

Overall, the decision curve analysis complements the calibration findings by showing that improved risk estimation accuracy post-recalibration leads to tangible gains in expected clinical benefit–particularly in threshold ranges where resource constraints or intervention harms must be carefully weighed. This reinforces the importance of recalibrating prediction models before deployment in new populations with different risk distributions.

### Stratified analysis of model performance

To assess how the original and recalibrated eFalls models performed across different clinical strata, two binary indicators were used: ML_stratifier, flagging individuals with depression, general mental health concerns, self-harm, severe mental illness, or stress; and Learning Disability, identified via diagnostic codes. As detailed in Section 2.2, these stratifiers partitioned the Trust cohort into subgroups: MH_only, LD_only, MH_and_LD, No_MH_No_LD, and the broader aggregates Any_MH, Any_LD, No_MH, and No_LD.

Both the original and recalibrated eFalls models were applied uniformly across these groups. Recalibration was performed once using the full working-age cohort to reflect realistic deployment, avoiding subgroup-specific fitting. Calibration and discrimination results for each subgroup are shown in Table 5.

### Analysis with the sex-based cohorts

Here we examined performance stratified by sex. Event prevalence was slightly higher among females (2.37%) compared to males (1.74%). We applied the same models–i.e., the original eFalls model and the recalibrated model that was fitted once on the entire Trust cohort. We did not retrain or recalibrate separately within each subgroup. This approach reflects a realistic deployment setting, where a single, system-wide recalibration is preferred for consistency and simplicity. Table 4 summarises the calibration and discrimination metrics obtained for each subgroup using this strategy.

**Table 4:** Performance metrics of the fall risk prediction model across sex-based subgroups and the whole cohort. Both original and recalibrated model outputs are shown.

Group	N	Fall Count	Fall Rate	Orig Slope	Orig CITL	Orig O/E	Orig AUC	Recal Slope	Recal CITL	Recal O/E	Recal AUC
Male	15464	269	1.74%	1.304	1.335	1.206	0.783	1.000	0.000	1.000	0.783
Female	16946	402	2.37%	1.235	1.699	2.060	0.784	1.000	0.000	1.000	0.784
Whole	32410	671	2.07%	1.223	1.358	1.604	0.777	1.000	0.000	1.000	0.777

Before recalibration, both subgroups exhibited evidence of systematic underprediction of risk, but the extent differed in magnitude. For males, the observed-to-expected (O/E) ratio was 1.206, indicating that the number of falls was approximately 20% higher than expected under the original model. In contrast, the O/E ratio for females was markedly higher at 2.060, meaning the model underestimated falls by more than a factor of two in this subgroup. This disparity is mirrored in the calibration-in-the-large (CITL) estimates: 1.335 for males and 1.699 for females. These values highlight that, on average, predicted risks were too low, with miscalibration being more pronounced for females. The calibration slopes also pointed to under-dispersion in both groups (male slope = 1.304; female slope = 1.235). In this scenario, the spread of predicted probabilities was too narrow, compressing the risk distribution and failing to sufficiently differentiate between lower- and higher-risk individuals. Such behaviour is more problematic in the female subgroup, where both under-dispersion and strong underprediction (high CITL and O/E) coincided.

When compared to the whole cohort (CITL = 1.358, slope = 1.223, O/E = 1.604), the sex-based results show a consistent pattern: males exhibited slightly better calibration than the full cohort, whereas females performed worse, with higher O/E and CITL values. This suggests that the model underestimates fall risk in both sexes, but more severely so in women.

Despite these calibration deficiencies, discrimination remained relatively robust and nearly identical across sexes, with C-statistics of 0.783 for males and 0.784 for females. These values are slightly higher than that observed for the whole cohort (0.777), suggesting that the model’s ability to rank individuals by risk is preserved and even marginally improved within sex-specific subgroups. This indicates that while the absolute probabilities assigned to females are substantially biased downward, the relative ordering of risk across individuals remains accurate and consistent with, or better than, the overall population.

Following logistic recalibration, both sex subgroups achieved near-ideal calibration. Slopes were corrected to 1.000 and CITL values aligned close to zero, while O/E ratios converged to 1.000. These adjustments show that post-hoc recalibration was highly effective in addressing the systematic underestimation of falls in both groups. Importantly, however, recalibration left discrimination unchanged, with AUCs remaining at 0.783 and 0.784 for males and females, respectively.

Taken together, these findings suggest that the original model underestimated fall risk in both sexes, but this miscalibration was greater for females. While recalibration corrects average calibration metrics, the persistence of sex-based discrepancies in baseline event rates implies that sex-specific risk factors may not be fully captured by the existing predictor set.

### Subgroup performance analysis by mental health and learning disability status

Table 5 summarises key performance metrics before and after recalibration, across several stratified subgroups defined by mental health conditions (denoted as MH) and learning disability (denoted as LD).

**Table 5:** Performance metrics of the fall risk prediction model across key subgroups defined by mental health (ML_stratifier) and learning disability (LD) status. Both original and recalibrated model outputs are shown.

Group	N	Fall Count	Fall Rate	Orig Slope	Orig CITL	Orig O/E	Orig AUC	Recal Slope	Recal CITL	Recal O/E	Recal AUC
MH_only	10828	243	2.24%	1.228	1.040	1.236	0.760	1.000	0.000	1.000	0.760
LD_only	1467	129	8.79%	0.763	0.986	5.720	0.696	1.000	0.000	1.000	0.696
MH_and_LD	304	34	11.18%	0.964	1.881	5.929	0.739	1.000	0.000	1.000	0.739
No_MH_No_LD	19811	263	1.33%	1.494	2.373	1.368	0.815	1.000	0.000	1.000	0.815
Any_MH	11132	277	2.49%	1.193	1.025	1.369	0.753	1.000	0.000	1.000	0.753
Any_LD	1771	163	9.20%	0.803	1.170	5.762	0.707	1.000	0.000	1.000	0.707
No_MH	21278	394	1.85%	1.371	2.156	1.824	0.792	1.000	0.000	1.000	0.792
No_LD	30639	506	1.65%	1.304	1.452	1.302	0.795	1.000	0.000	1.000	0.795
Whole	32410	671	2.07%	1.223	1.358	1.604	0.777	1.000	0.000	1.000	0.777

#### Analysis with the LD-based cohorts

In the learning disability (LD) related subgroups, we observe consistent evidence of systematic underprediction of fall risk. All three cohorts—LD_only, Any_LD, and MH_and_LD—show exceptionally high observed-to-expected (O/E) ratios of 5.720, 5.762, and 5.929, respectively. These values indicate that the number of observed fall events is more than five times higher than what the model predicts, revealing a profound mismatch in predicted risk.

Interestingly, the corresponding calibration-in-the-large (CITL) values in these subgroups are less extreme (0.986, 1.170, and 1.881), which might superficially suggest that the model is reasonably well-calibrated in terms of average log-odds. However, this stands in stark contrast to the extreme O/E ratios. The apparent discrepancy suggests that the model does not allocate enough predicted probability to individuals who go on to experience a fall, likely because predicted risks are too concentrated at the lower end of the probability spectrum. As such, even a seemingly modest CITL can mask significant miscalibration when the true event burden is heavily underestimated—a situation especially pertinent in high-risk or marginalised populations like those with learning disabilities.

Supporting this, all three LD-related groups show calibration slopes below 1 (0.763, 0.803, and 0.964), indicating over-dispersion in the model’s predicted probabilities. In such cases, predictions are spread too far from the average: low predicted risks are pushed lower, and high predicted risks are pushed higher. While this might theoretically suggest overestimation for high-risk individuals, the practical impact depends heavily on the actual distribution of predicted probabilities. If the majority of predictions in the LD-related cohorts lie in the low-to-moderate range (as is often the case when model outputs rarely exceed 10–25%), further reducing these already-low values leads to even greater underprediction. This dynamic helps explain how a near-zero CITL can coexist with a very high O/E ratio and a calibration slope less than 1.

When comparing these LD-related subgroups against the full cohort (Whole), several important trends emerge. First, although the CITL for the full cohort is already elevated at 1.358—suggesting overall underestimation—it remains lower than the CITL observed in the MH_and_LD group (1.881), and not dramatically different from the LD-only group (0.986). Yet, in contrast to the relatively modest CITL values, the observed-to-expected (O/E) ratios for all LD subgroups (LD_only: 5.720, MH_and_LD: 5.929, Any_LD: 5.762) are strikingly higher than that of the full cohort (1.604), indicating that underprediction is substantially more severe for those with learning disabilities. This divergence between CITL and O/E—as discussed earlier—highlights the calibration challenge posed by these subgroups: the average prediction (CITL) may appear superficially acceptable, but the total number of events remains dramatically underpredicted, pointing to a failure to assign high enough probabilities to those most at risk.

In terms of discrimination, these LD-related subgroups also exhibit poorer performance compared to the whole population. The C-statistic for the Whole cohort is 0.777, reflecting moderate-to-good discriminatory ability. In contrast, the LD subgroups show consistently lower AUCs: LD_only at 0.696, MH_and_LD at 0.739, and Any_LD at 0.707. These values suggest that the model is less capable of correctly ranking individuals by fall risk within the LD populations. Such a drop in AUC is likely due to a combination of factors: limited sample sizes, different risk factor profiles, or inadequate representation of LD-specific predictors in the original model training data.

While the original model exhibited substantial calibration issues in the LD-related subgroups, the recalibrated version notably improved calibration metrics across the board. All three subgroups–LD_only, Any_LD, and MH_and_LD–achieved ideal recalibration slopes of 1.000 and recalibrated CITL values close to zero, suggesting that the logistic post-hoc adjustment effectively corrected both the overall intercept and the scale of predicted probabilities. The observed-to-expected (O/E) ratios also converged to 1.000, indicating that the total number of observed events now closely aligns with expected values. These improvements confirm that logistic recalibration is capable of resolving systematic underprediction, at least in terms of average calibration metrics. However, it is important to note that recalibration does not improve discrimination: the AUC values remained unchanged at 0.696, 0.707, and 0.739, respectively. This implies that while the recalibrated model may better estimate the overall probability of falling, it still struggles to rank individuals correctly within these LD-related groups. Thus, recalibration addresses calibration deficiencies but does not fully resolve the performance gap in these high-risk subpopulations, which might suggest structural model adaptation or inclusion of LD-specific predictors may be necessary for further improvement.

Altogether, these patterns suggest that the model struggles to adequately elevate risk scores for LD patients, not very well reflecting their true underlying vulnerability. This reinforces the need for population-specific adjustments–such as subgroup-aware recalibration or enrichment with features more predictive within the LD population–to mitigate systematic underestimation in these high-risk groups.

#### Analysis with the MH-based cohorts

We next examine the model’s performance across subgroups defined by the presence of mental health (MH) difficulties. These include: MH_only (individuals with one or more mental health conditions but without learning disability), Any_MH (all individuals with any mental health indicator, including those with comorbid learning disability), and MH_and_LD (those with both mental health conditions and learning disability).

In terms of observed-to-expected (O/E) ratios, all MH-related subgroups exhibit values greater than 1.0: MH_only at 1.236, Any_MH at 1.369, and MH_and_LD at 5.929. These results indicate that the model systematically underestimates fall risk in individuals with mental health difficulties–especially those who also have learning disabilities. Notably, the underprediction is much more extreme in the MH_and_LD subgroup, suggesting that learning disability exerts a disproportionately negative impact on model calibration, even within the broader MH cohort.

This divergence is further supported by the calibration-in-the-large (CITL) values: 1.040 for MH_only, 1.025 for Any_MH, and 1.881 for MH_and_LD. While all three suggest average underprediction, the CITL for MH_and_LD is substantially higher, again pointing to deeper miscalibration when learning disability is present. Interestingly, although Any_MH includes all individuals with MH conditions (including those with LD), its performance appears to largely reflect the much larger MH_only component. This further underscores how a relatively small number of individuals with LD (e.g., N = 304 for MH_and_LD) can dramatically affect model performance when disproportionately mispredicted.

The calibration slopes show a mixed picture. The MH_only and Any_MH subgroups exhibit slopes slightly above 1 (1.228 and 1.193, respectively), indicating under-dispersion, where predicted probabilities are overly compressed around the mean. This results in reduced contrast between low and high-risk individuals. In contrast, the MH_and_LD group has a slope of 0.964–slightly below 1–indicating mild over-dispersion. This suggests that risk predictions for individuals with both MH and LD are not only miscalibrated but also overly spread out, potentially amplifying prediction errors in both directions. Taken together, these patterns highlight that the combination of MH and LD creates a particularly difficult calibration scenario.

When compared to the Whole cohort (CITL = 1.358, O/E = 1.604, Slope = 1.223, AUC = 0.777), the MH_only and Any_MH subgroups perform only slightly worse, both in calibration and discrimination. The C-statistics for these groups are 0.760 and 0.753, respectively–only modestly lower than the overall model. However, the MH_and_LD subgroup again stands out, with a substantially lower AUC of 0.739, suggesting that combining MH and LD conditions substantially erodes the model’s ability to rank individuals by true risk. This may reflect not only greater clinical complexity but also an absence of sufficiently predictive features for this high-risk intersectional group.

Finally, recalibration using the full-cohort model greatly improved calibration metrics across all three MH-based groups. In each case, the CITL and slope were corrected to approximately 0 and 1, respectively, showing that post-hoc adjustment of the predicted probabilities can realign average risk and scale. However, discrimination–as expected–remained unchanged, indicating the limits of recalibration in addressing deeper structural or feature-related issues.

**Summary of Stratified Subgroup Findings.**

Taken together, the stratified analysis across both mental health and learning disability subgroups reveals a consistent trend: model calibration and discrimination degrade most substantially in the presence of learning disability, either alone or in combination with mental health difficulties. The MH-only subgroup showed only mild underprediction and relatively strong discrimination (AUC = 0.760), suggesting that the model retains reasonable performance for individuals with mental health needs in the absence of LD. However, once LD is present–particularly in the MH_and_LD group–both calibration and discrimination suffer markedly, with extreme O/E ratios (e.g., 5.929) and diminished AUC (0.739). In contrast, groups without LD consistently show better alignment with the model’s overall performance on the full Trust cohort. These findings indicate that while mental health conditions introduce modest challenges to predictive accuracy, learning disability is a stronger disruptor of model validity. Addressing this disparity is essential to ensuring equitable and reliable risk prediction in vulnerable subpopulations.

### Discussions

#### Positioning relative to NICE guideline NG249

The National Institute for Health and Care Excellence (NICE) provides evidence-based recommendations for health and social care in England and Wales. The recently updated NICE guideline on falls prevention (NG249) recommends against routine use of standalone falls risk prediction tools in older adults, citing limited evidence that such tools improve outcomes beyond comprehensive multifactorial assessment. This recommendation reflects the current evidence base, in which robust and widely validated falls prediction tools remain limited. We emphasise that the present study does not advocate replacing clinical judgement or established assessment pathways with automated prediction models.

Rather, the *eFalls* framework represents an electronic health record (EHR)-embedded risk stratification tool operating at a system level. In large populations where universal in-depth assessment may be impractical, such models may support prioritisation of clinical review or targeted preventive assessment. In this context, the model functions as a triage-support mechanism rather than a deterministic decision tool. Our study therefore contributes new evidence by exploring the feasibility of a system-level, EHR-embedded falls risk stratification approach rather than proposing a standalone clinical decision tool.

In our working-age mental health cohort, the model achieved a C-statistic of 0.777 and demonstrated good calibration following simple recalibration. C-statistic in the range of 0.7–0.8 is commonly regarded as acceptable discrimination^18^. Consistent with NICE guidance, any implementation of such models should remain locally validated, integrated within multifactorial assessment pathways, and used to complement–rather than replace–clinical decision-making.

#### Interpretation of declines in discrimination

The reduction in discrimination from 0.825 in the older development cohort to 0.777 in the working-age mental health population, and further to 0.696 in the learning disability subgroup, warrants careful interpretation. There is no universally accepted discrimination threshold that mandates redevelopment of a prediction model. However, discrimination in the range of 0.7–0.8 is commonly regarded as acceptable^18^. By contrast, C-statistics in the range of 0.5–0.7 are commonly interpreted as indicating poor discrimination, suggesting limited ability to distinguish between individuals with and without the outcome^18^.

In the overall Trust cohort (AUC = 0.777), discrimination remains within the range generally considered acceptable for population-level risk stratification. In contrast, the lower discrimination observed among individuals with learning disabilities (AUC = 0.696) suggests that recalibration alone may be insufficient and that structural model extension or the inclusion of learning-disability-specific predictors may be required to achieve equitable performance. These findings highlight that recalibration can correct systematic probability misalignment but does not address deeper differences in predictor–outcome relationships across subpopulations.

#### Potential role of age-specific recalibration

One potential contributor to the observed underprediction may be differences in the age–risk relationship between the original older development cohort and the younger validation population examined in this study. The original *eFalls* model was developed in adults aged *≥*65 years, a population in which fall risk is substantially higher and may increase non-linearly with advancing age. In contrast, the present validation cohort comprises working-age adults. As a result, the age distribution and baseline risk structure differ markedly between the two populations.

When prediction models are transported across populations with different age distributions and baseline risks, the estimated age coefficient may no longer accurately capture the relationship between age and outcome risk. In particular, risk gradients observed in older populations may not translate proportionally to younger cohorts, potentially contributing to systematic underprediction even when other predictors remain relevant. In addition, because the age coefficient was estimated using an older development cohort, it may partially reflect risk patterns specific to older populations. When applied to younger cohorts, this coefficient may not fully capture differences in how fall risk varies across age ranges, potentially contributing to systematic miscalibration.

An alternative updating strategy could therefore involve selectively refitting or updating the age coefficient while retaining other regression parameters, rather than applying a global intercept-and-slope recalibration. Such targeted coefficient updating, sometimes referred to as partial model revision, may allow improved transportability across age-divergent populations while preserving the structure of the original model. However, implementation would require access to the full original penalised model specification and careful safeguards against overfitting in the external dataset.

Importantly, the present findings do not alter the intended use of the *eFalls* model in older populations, where it was originally developed and validated. Rather, they highlight that when applying prediction models beyond their original age range, recalibration and careful evaluation of age-related effects are essential. Exploration of age-specific updating strategies, including empirical evaluation of whether updating the age coefficient alone improves calibration, represents an important direction for future work.

## Conclusion

This study provides the first clinical validation of the *eFalls* model in a working-age adult population within UK mental health and learning disability services. Despite being originally developed for adults aged 65 years and older, the model retained good discrimination (C-statistic = 0.777) when applied to the cohort of a large integrated teaching NHS foundation trust in the north of England. However, systematic underestimation of risk was evident before recalibration (CITL = 1.36; O/E = 1.60), highlighting differences in baseline risk and predictor–outcome relationships between older and younger populations.

Simple logistic recalibration of the model intercept and slope corrected this misalignment, restoring good calibration (CITL *≈* 0; O/E *≈* 1) without altering discrimination. These results demonstrate that the *eFalls* framework can be transported across populations when appropriate recalibration is performed. Importantly, these findings do not alter the intended use of the model in older populations, where it was originally developed and validated.

Subgroup analyses revealed notable heterogeneity in performance. While the model performed comparably across sex and mental health–only groups, discrimination and calibration degraded among individuals with learning disabilities, particularly those with coexisting mental health conditions. This pattern suggests that learning disability may represent an important axis of prediction disparity and that recalibration alone may be insufficient to address subgroup-specific differences in predictor–outcome relationships. Future model adaptations may therefore benefit from incorporating learning-disability-specific predictors or interaction terms.

Overall, this validation demonstrates the feasibility of extending fall risk prediction to working-age adults receiving mental health services. When carefully recalibrated and locally evaluated, EHR-based models such as *eFalls* may support system-level risk stratification and prioritisation of clinical review, provided they are used to complement rather than replace multifactorial clinical assessment.

## Supplementary Information

Supplementary Information 1.

## Data Availability

Data are available on reasonable request.
