# Supplementary material for: Extending eFall risk prediction to working-age adults within mental health and learning disability services: a clinical validation study
Source: Sci Rep. 2026 Aug 10;16:37294. doi: 10.1038/s41598-026-51298-0 (PMC13745281; doi:10.1038/s41598-026-51298-0)
Supplement: Supplementary file 1 [file 41598_2026_51298_MOESM1_ESM.pdf]

Supplementary Information

Supplementary Table S1. Predictors and regression coefficients from the final penalised *eFalls* model

The table below lists the predictor variables and regression coefficients from the final penalised logistic regression model used to compute the *eFalls* linear predictor. These coefficients were applied without modification in the present external validation study. Reference categories correspond to the baseline levels used in the original *eFalls* model.

Predictor	Coefficient
**Demographic variables**	
Age (years)	0.0415506
Medication count (Polypharmacy): $\ln((\text{Medication count}+1)/10)$	0.3296295
Gender	
Male	Reference
Female	-0.303708
BMI category	
Underweight	0.4896735
Normal	0.2394177
Overweight	Reference
Obese	-0.0411134
Missing	-0.1451981
Smoking	
Ex/never	Reference
Current	0.0684529
Alcohol consumption	
Harmful drinking	0.4164064
Higher risk drinking	0.1549725
Lower risk drinking	Reference
Previous higher risk/harmful drinking	0.0849676
Zero alcohol	0.0070124
Missing	-0.0679367
**Clinical conditions**	
Abdominal pain	-0.0641861
Activity limitation	0.0475092
Anaemia and haematinic deficiency	0.1733029
Asthma	0.0816046
Atrial fibrillation	0.1519654
Back pain	0.0498699
Bone disease	-0.0267845
Cancer	0.0301854
Cognitive impairment	0.1472251
COPD	0.0399560
Dementia	0.1038111
Depression	0.1633415
Diabetes mellitus	0.0373911
Dizziness	0.0198363
Dressing and grooming problems	0.4532777
Faecal incontinence	-0.0717910
Falls	0.3009161
Fatigue	-0.0635057
Foot problems	0.0282736
Fracture	0.1957923
Fragility fracture	0.2031303
General mental health problems	0.0991068
Headache	-0.0149365
Hearing impairment	-0.0168728
Heart failure	-0.0190399

Continued on next page

**Predictor**	**Coefficient**
Housebound	0.2549983
Hypertension	-0.0318888
Hypotension or syncope	0.0778591
Inflammatory arthritis	0.0586785
Inflammatory bowel disease	0.0145135
Liver problems	0.3803626
Meal preparation problems	-0.1400240
Medication management	0.8030273
Memory concerns	0.2601186
Mobility problems	-0.1310067
Mono or hemiparesis	0.1020457
Motor neurone disease	-0.1209976
Musculoskeletal problems	0.0419361
Osteoarthritis	0.0634073
Osteoporosis	0.1276254
Palliative care	-0.2353552
Parkinsonism and tremor	0.2312839
Peptic ulcer disease	0.0056687
Peripheral neuropathy	0.0335789
Peripheral vascular disease	0.0173065
Requirement for care	-0.2177301
Respiratory disease	0.0132757
Seizures	0.2571899
Self-harm	0.1461241
Severe mental illness	0.0676153
Skin ulcer	0.0746926
Sleep problems	0.0056967
Social vulnerability	0.0495075
Stress	-0.0200494
Stroke	0.0788542
Thyroid problems	-0.0273864
Urinary incontinence	0.0345173
Urinary system disease	0.0119309
Visual impairment	0.0233200
Washing and bathing	-0.1118824
Weakness	-0.0589464
Weight loss	0.0301414
Constant (Intercept)	-5.954459
